# A randomized controlled experiment testing the use of virtual reality to trigger cigarette craving in people who smoke

**DOI:** 10.1038/s41598-024-70113-2

**Published:** 2024-08-21

**Authors:** Aitor Rovira, Sinéad Lambe, Helen Beckwith, Memoona Ahmed, Felicity Hudson, Phoebe Haynes, Chun-Jou Yu, Kira Williams, Simone Saidel, Ellen Iredale, Sapphira McBride, Felicity Waite, Xueni Pan, Daniel Freeman

**Affiliations:** 1 Department of Experimental Psychology, University of Oxford, Oxford, UK; 2 Oxford Health NHS Foundation Trust, Oxford, UK; 3 Goldsmiths University, London, UK

**Keywords:** Cigarette craving, Virtual reality, Psychology, Human behaviour

## Abstract

Automated delivery of therapy in virtual reality (VR) has the potential to be used for smoking cessation. Most obviously, it could be used to practise and establish alternative reactions to smoking cues. The first step in treatment development is to show that VR environments can trigger sufficient cravings in smokers. We evaluated a new VR public house outdoor scenario with 100 individuals who smoked daily. Participants were randomly assigned to the VR scenario with smoking cues or a neutral experience in VR. The VR experiences were presented in a standalone VR headset. Before and after VR, we collected self-reported craving scores for cigarettes and alcohol using the Tobacco Craving Questionnaire (TCQ) and visual analogue scales (VAS). Physiological data were also collected. Compared to the neutral condition, exposure to the smoking cues led to a large increase in craving for a cigarette (TCQ β = 11.44, p < 0.0001, Cohen’s d = 1.10) and also a moderate increase in craving for alcohol $(\upbeta =0.7,\text{ p}=0.017,\text{ d}=0.50)$. There were no significant physiological differences between the two conditions. These results provide good evidence that VR experiences can elicit strong craving for cigarettes. The programming can be part of developing a new VR cognitive therapy to help people reduce smoking.

## Introduction

People often smoke in response to specific cues such as seeing a cigarette, ashtray, or matches^1,2^. Hence exposure to smoking cues is an important step in therapies designed to build resilience to craving^3^. Presentation of smoking cues within virtual reality (VR) has been shown to elicit cigarette craving^4^. There are two key advantages of use of VR. First, multiple different smoking cues and scenarios, graduated in difficulty, can be easily presented and there are no actual cigarettes present to smoke and reinforce the established response. Second, it is now possible to automate delivery of therapy within VR^5^. We have successfully piloted with thirteen smokers a VR smoking environment delivered in the new generation of standalone VR headsets^6^. In this paper, we report a definitive test of this environment as the first step in developing a new VR therapy for smoking cessation.

One important reason that prevents people who smoke from successfully quitting is the difficulty of not responding to everyday smoking cues^1^. Smoking cues can be pervasive in daily lives and exposure to these cues is a predictor of smoking^7^. These cues may be specific items related to smoking, such as ashtrays and cigarette butts, and general environments where people usually smoke, such as a bar, and can include time-related events such as a morning coffee routine^8^. Exposure to smoking cues elicits craving, and craving has been identified as the mediator that leads to smoking^9–11^. Drinking alcohol is another well-recognised cue for smoking^12^ and people who drink alcohol are more likely to smoke too^13^.

Exposure to smoking cues triggering craving for a cigarette is a well-replicated phenomenon^1,14^. The results are consistent across different means of presentation, including pictures^15^, video^16^, feature films^17^, and VR^18^. VR clearly provides a higher degree of experimental control compared to studying occurrences of smoking in a natural setup and also provides a higher degree of immersion and interaction than 2D technologies, in which a typical setup offers a reduced field of view and participants are simply spectators^19^. Furthermore, VR allows the placement of people in a surrounding virtual environment that may be associated to smoking, thus triggering craving from a broad contextual cue^20^.

In a systematic review of 18 studies that involved 541 smokers, it has been shown that VR presentation of cues can produce a large triggering of craving (Cohen’s d = 1.0)^21^. In the largest study to date we wanted to show that similar effects can be produced from delivery of VR scenes in the new generation of standalone headsets. This could then form the basis of the development of a new cognitive intervention for smoking cessation. There is extensive evidence in the literature that exposure to smoking cues in VR triggers craving^18,22, 23^. However, only a few papers have reported the results of randomised control tests of the use of immersive VR technologies so far. A number of studies used VR to expose smokers to cues^24^ or used VR to try to improve the results of an approach bias modification approach^25^. Other studies used more limited technologies such as 360-degree videos^26^ and Second Life^27^. These studies have taken different approaches in experimental design or the experimental setup, making it difficult to compare their results. None have tested long-term effects.

Subjective measurements are the most common way to assess cravings. A small number of studies have also supplemented self-report with objective measurements such as physiological data. For example, it has been suggested that craving can be a predictor of physiological arousal^28^, and skin conductance has been shown to increase after exposure to smoking cues^29^. Therefore we also included physiological measures in our test.

In our pilot study, simple pre and post testing with 13 smokers indicated that our new VR environment may increase smoking craving^6^. In this paper, we present the results of a randomised controlled study with 100 participants using the same VR smoking cue scenario. We collected self-reported measurements through questionnaires and physiological data related to heart rate and skin conductance.

## Methods

### Experimental design

The study was a between-subject experiment in which we carried out a between group comparison of smoking craving scores after going through a VR experience, either a neutral environment or a scenario depicting potential smoking cues. Participants were randomly allocated to an experimental group using the online tool Sealed Envelope (https://www.sealedenvelope.com/). Ethics approval was granted by the University of Oxford Central University Research Ethics Committee (reference R81586/RE001). All research was performed in accordance with relevant guidelines/regulations, and written informed consent was provided by all participants.

### Participants

Participants were recruited through advertisements on social media and local radio stations. The inclusion criteria were: over 18 years old and smoke a minimum of 10 cigarettes per day. The exclusion criteria were: photosensitive epilepsy; significant visual, auditory, or balance impairment; insufficient comprehension of English to complete study measures; using nicotine replacements; primary diagnosis of another substance dependency; or medication that reduces nicotine cravings (e.g. Bupropion).

### Measures

The main outcome of the study was the score from the Tobacco Craving Questionnaire^30^. It is a 12-item short version of the long 47-item questionnaire. The long version is validated and reported as being reliable for research^31^ and the short version has similar internal consistency to the original version. It assesses craving for a cigarette at the time of filling it out, with answers on a 1 (strongly disagree) to 7 (strongly agree) Likert scale. Participants filled out this questionnaire before and after the VR experience. Scores can range from 12 to 84. Higher scores indicate greater craving for a cigarette.

As additional outcomes, participants provided subjective scores of their current cravings of cigarettes and alcohol using two visual analogue scales (VAS), with answers from 0 (Do not want one at all) to 10 (Extremely want one). Participants provided their current cravings before and after the VR experience. Higher scores indicate greater craving. Although internal consistency cannot be checked due to being just a single item, the use of VAS has been an increasingly popular method to quantify subjective experiences (e.g. pain^32^), and has been found to be a valid way to measure intensity of cigarette craving^33^.

At baseline participants completed the Heaviness of Smoking Index (HSI)^34^, which is a 2-item questionnaire to assess how much a person smokes, including the questions “How many cigarettes do you typically smoke per day?” and “How soon after you wake up do you have your first cigarette?” Answers were given as categorical numbers. These two measurements have been shown to be fairly reliable when used either separately or together^35^.

At baseline participants completed the AUDIT-C^36^, which is a three-item questionnaire to assess average alcohol use. Multiple studies have validated this questionnaire^37^.

Electrodermal activity and heart rate were recorded during baseline and the VR experience with the use of an Empatica E4 wristband (https://www.empatica.com/en-gb/research/e4/). Data included two pairs of inter beat interval (IBI) and electrodermal activity (EDA) files, one pair for baseline and the other recorded during the VR experience.

### VR scenarios

We used the Meta Quest 2 VR headset in standalone mode for all the VR sessions. That means we used the VR headset without using a computer to run the simulation. There were two scenarios, one for the experimental group and one for the control group. Each lasted three minutes. In both scenarios participants sat down for the entire duration of the experience.

**Figure 1:**
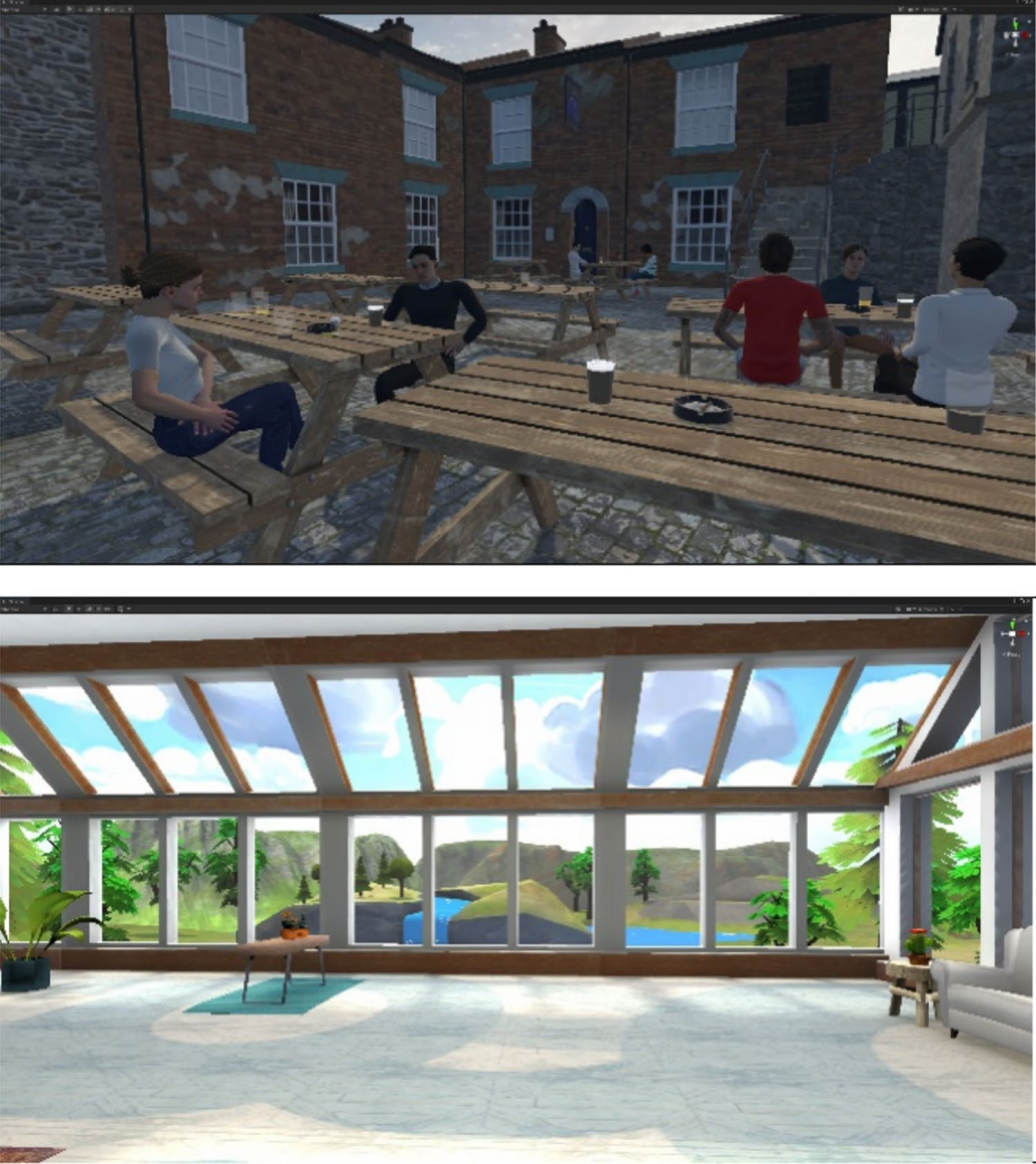
Screenshots of the VR scenarios taken from the initial perspective of the participant (1) the beer garden; (2) the room in the neutral environment. Images created in Unity 2020.3.3f1 (https://unity.com/releases/editor/whats-new/2022.3.3).

In the experimental group, participants were placed in an environment that resembled a British pub outdoor space^6^ (see Fig. 1). There were several people sitting around as on a typical warm sunny day. There were several items related to smoking cues in the scenario—pint glasses, ashtrays with cigarette butts, one of them half extinguished and still releasing a trail of smoke. On the bench next to the participant, two virtual characters were chatting. Over time their discussion turned towards cigarettes and how hard it is to quit smoking. At the end of the scenario, one of the characters turned their head towards the participant and asked them directly if they wanted a cigarette.

In the control group, participants visited a neutral environment in a modern house with wide windows, similar to the welcome room described in^38^ (see Fig. 1). The landscape outside included different types of vegetation, a water stream, and clear blue sky. The environment included quiet background music. This environment did not contain any smoking cues.

No other hardware was required besides the VR headset, the Empatica E4 wristband, and a smartphone to record the physiological data.

### Experimental procedures

Participants were asked to refrain from smoking 30 min before coming to the VR lab. Upon arrival, they were met at the reception area by a researcher who guided them to the VR lab. Once in the lab, they were asked to confirm that they had read the information sheet at least 24 h prior to the VR session and if willing to participate, to sign a consent form. After agreeing to participate, they filled out the AUDIT-C, the Tobacco Craving Questionnaire, and the visual analogue scales to obtain baseline measurements for the initial craving scores.

When they completed the questionnaires, they were randomised and allocated to the experimental condition they were instructed to remain seated for the rest of the session. A researcher helped them put the Empatica E4 wristband on their dominant arm and recorded two minutes of physiological data as a baseline. After that, participants put the VR headset on, the researcher made sure that vision was clear and the headset had been adjusted to the participant’s comfort, the Empatica E4 started recording data once again, and the VR experience started.

After the VR experience ended, the researcher helped them to take the VR headset off and were asked to fill out the Tobacco Craving Questionnaire and the visual analogue scales again. Participants were compensated twenty pounds for their time.

### Data analysis

Analyses were conducted in R version 4.3.0^39^. The main outcome was the craving score on the TCQ after the VR experience. We carried out a linear regression analysis with experimental group (StudyCondition) as the independent variable and controlling for initial craving scores.

$$Tobacco \,Craving \,Questionnaire \,\left(TCQ\right)score \sim StudyCondition + TCQBaseline$$

The scores reported by participants on the two VASs (cigarettes and alcohol) were also analysed using linear regression. Similarly, we used group as the independent variable and controlling for initial craving scores. Linear regression analyses were carried out in R using the *lm* function. Effect sizes were summarised as Cohen’s d values calculated using the *cohen.d* function in R.

We tested scores for how much participants smoked (HSI) and drank (AUDIT-C) on average as possible moderators of the main outcome with the following equation:

$$TCQ \,score \sim StudyCondition + HSI+ AUDIT\_C+ StudyCondition * HSI + StudyCondition * AUDIT\_C$$

Electrodermal activity (EDA) data pre-processing and initial visual analysis was carried out using the Matlab-based tool Ledalab^40^. We carried out a visual inspection on each dataset to detect anomalies in the data. We discarded the data from participants if more than 50% of their data were zero on either the baseline or the VR experience dataset. We also discarded the data showing sudden jumps that were too abrupt to be attributed to a change in skin conductance and did not recover to the original level after a few seconds.

Data cleaning^41^ included data trimming, smoothing, and correction of artifacts originated from bad readings. We trimmed a few seconds in the beginning and in the end of the dataset as it was common that there were a few faulty readings at both ends. Trimming was done manually keeping the data from the moment the function looked stable. We smoothed out the data to remove high frequency noise using a filter with a Gauss window size 8. We also removed any isolated spike due to bad readings and we reconstructed the signal with either a linear or a Spline interpolation, depending on what was more suitable in each case. The sample rate was kept at the recording rate of 4 Hz.

We split the signal between the tonic and the phasic components using continuous decomposition analysis^40^. The tonic component provides an overall background level and tendency over time of the signal, while the phasic component contains the information about sudden peaks and changes. We then analysed both components separately. We looked at the mean and standard deviation in the tonic component during the VR experience relative to the baseline. For this, we divided both the mean and standard deviation obtained in the VR experience by the values obtained in the baseline. We also looked at the skin conductance level (SCL) as the gradient of the tonic component. In the phasic component, we studied the mean and standard deviation relative to baseline the same way we calculated it in the tonic component. We then compared these extracted features between experimental groups.

Regarding heart rate data, we were interested in the heart rate variability (HRV), calculated from the IBI data. These data are processed only when the two beats are detected, thus the number of samples varied between participants. The mean and standard deviation of the HRV used in the statistical analysis were also relative to the baseline values.

We analysed all the features extracted from both EDA and IBI signals in a linear regression with the experimental group as the sole independent variable.

## Results

30 male and 22 female participants were allocated to the experimental group. The average age was 39.12 (SD = 15.12). In the control group there were 27 male and 21 female participants, with an average age of 37.77 (SD = 14.49). No participants selected their gender as either ‘non-binary’ or ‘preferred not to say’. In the experimental group, the average HSI score was 3.33 (SD = 1.20) and the AUDIT-C score was 5 (SD = 3.01), and in the control group, the average HSI was 3.25 (SD = 0.84) and the average AUDIT-C score was 6.25 (SD = 2.97).

Table 1 shows the scores of the three questionnaire outcomes. All three scores obtained after the VR experience were statistically different between experimental groups. Compared to the neutral condition, the experimental group had large effect size increases in cigarette craving and a moderate increase in craving for alcohol. The Heaviness of Smoking Index reported during screening predicted cigarette craving after the VR session across both experimental groups (HSI, p < 0.0001) and alcohol use did not (AUDIT-C, p = 0.85). Looking within groups (i.e. pre to post changes), the experimental group increased in scores on the TCQ (p < 0.01), the VAS for cigarettes (p < 0.0001), and the VAS for alcohol (p < 0.001). The control group decreased in scores on the TCQ (p < 0.0001) and the VAS for cigarettes (p = 0.02) but did not significantly alter in alcohol craving (p = 0.18).

**Table 1:** Mean, std deviation and tests results of the craving outcome scores.

	Experimental group (n = 52)	Control group (n = 48)	Adjusted group difference (95% CI)	Effect Size (Cohen’s d)	p value
Cigarette craving (TCQ)					
Baseline	45.63 (17.17)	43.79 (11.80)	.	.	.
After VR	49.69 (20.24)	36.46 (14.65)	11.44 (7.27 to 15.60)	1.10	< 0.0001
Cigarette VAS					
Baseline	4.71 (2.50)	4.75 (1.99)	.	.	.
After VR	6.00 (3.00)	3.93 (2.90)	2.13 (1.27 to 2.99)	1.00	< 0.0001
Alcohol VAS					
Baseline	0.86 (1.71)	0.73 (1.16)	.	.	.
After VR	1.70 (2.43)	0.94 (1.54)	0.70 (0.13 to 1.27)	0.50	0.017

There were missing data from the physiological recordings. We had electrodermal activity (EDA) data from 71 participants (42 in the experimental group and 29 in the control group). Table 2 shows the mean, the standard deviation, and the results from the regression analyses of the different features extracted from the physiological data. The results did not show any statistically significant difference between experimental groups on any of the features extracted.

**Table 2:** Mean, standard deviation, and test results between experimental groups of the features extracted from the physiological data.

	Experimental group (n = 42)	Control group (n = 29)	Adjusted group difference (95% CI)	Effect size (Cohen’s d)	p value
Electrodermal activity (EDA)					
Tonic mean	1.42 (1.35)	1.26 (1.36)	0.24 (− 0.44 to 0.92)	0.17	0.49
Tonic std dev	0.61 (0.50)	0.75 (0.97)	− 0.14 (− 1.05 to 0.77)	0.07	0.76
Skin conductance level	− 0.38 * 10^–3^ (0.003)	0.82*10^–3^ (0.005)	− 0.001 (− 0.003 to 0.001)	0.32	0.20
Phasic mean	0.96 (0.78)	1.89 (2.70)	− 0.39 (− 2.24 to 1.47)	0.10	0.68
Phasic std dev	1.37 (1.37)	1.52 (1.91)	− 2.00 (− 6.38 to 2.39)	0.22	0.37
Heart rate variability (HRV)					
IBI mean	1.08 (0.17)	1.09 (0.14)	− 0.01 (− 0.04 to 0.02)	0.00	0.41
IBI std dev	0.77 (0.38)	0.86 (0.6&)	− 0.10 (− 0.28 to 0.09)	0.00	0.31

## Discussion

We conducted the largest experimental test of whether VR simulations can produce craving for cigarettes in people who smoke regularly. Importantly the test used the latest standalone headset without use of an external computer. The VR public house scene produced a large increase in cigarette craving compared to a neutral VR scene. The Cohen’s d for cigarette craving was 1.1, which is similar to the effect size reported in a meta-analysis of studies focused on cue-induced craving in VR^21^. The VR pub experience led to significantly increased levels of craving from before to after immersion (i.e., there was a within group effect), but it should be noted when considering the magnitude of the between groups effect that there was also a significant reduction in cigarette craving in the neutral experience. This reduction may perhaps be explained by the use of VR technology being interesting for the participant and hence distracting from cravings. Furthermore, it is possible that the virtual environment, which had windows with views to a natural landscape, might have been found calming like a relaxation nature scene^42^﻿. The VR pub scene also led to an increase in the smokers in craving for alcohol. The results once again show how VR can induce similar responses to real-world environments. Our VR pub scene could form the basis for the development of a smoking cessation therapy.

We tested whether level of smoking and alcohol consumption affected responses. Neither had a differential affect by the type of VR scene. However, people who reported smoking a greater number of cigarettes had higher levels of cigarette craving in both the VR scene and the neutral scene. In contrast, level of alcohol use did not predict level of cigarette craving in VR. This further validates the use of VR, since it shows that, as would be expected, cravings elicited in VR are affected by a person’s severity of smoking (but not alcohol use).

Regarding the physiological information collected, we explored different features from electrodermal and heart rate data that could be related to craving. The tonic component in the electrodermal data could reveal an overall increase in anxiety. We analysed the mean and standard deviation of this signal relative to the data recorded during baseline. We also looked at skin conductance by calculating the gradient of the linear regression. Electrodermal values naturally change over time, so we predicted that in the experimental group there would be a significantly higher number of participants with a positive slope compared to the control group. We did not find evidence that any feature was statistically different between the randomised groups. Table 2 shows that the values are in the decimals, so we speculate that the signal-to-noise ratio was possibly close to zero dB. Skin conductance values were low. That means that the overall tonic values did not change to any great degree for any participant. Analysing the tonic driver might be more meaningful in longer experiences than three minutes.

The phasic signal is a marker of how participants respond to specific events during the experience. The VR pub scenario contained several smoking cues. We were interested in looking at whether these cues could trigger craving in very specific timestamps thus showing a spike in the data. We analysed the mean and standard deviation of the phasic driver, but the results did not show any difference in the data between the two groups. Data from an accelerometer can provide the information to see if a change in the electrodermal data comes from the movement of limbs. Additionally, electrodermal values can change when people talk, so a voice detection algorithm could be helpful. We finally studied the inter-beat interval to look for statistical differences in the heart rate variability. Again, we looked at the mean and standard deviation relative to baseline for each participant. The results did not show any statistical differences in this case either. Given the strong findings for subjective craving, it is plausible that we did not assess the most useful physiological information to detect it.

The Empatica E4 device has been validated^43,44^﻿. However, there were missing data. Researchers need procedures in place for setting it up correctly and to expect that the signal might change if the wristband moves. Recordings were three minutes long. Longer sessions would have provided better estimations of the tonic driver and the overall skin conductance level over time. On the other hand, the phasic signal detecting peaks should not have been too affected by the length of the recording. However, it should be kept in mind that changes in the phasic driver induced by stimuli will be reflected in the signal a few seconds later, between one and five seconds^40^. Our scenario with smoking cues ended with one of the characters looking directly at the participant and offering a cigarette. Recording was stopped right at that moment, whereas it should have carried on longer to capture the response. For the phasic driver, it is important to make an estimation of the signal-to-noise ratio (SNR) to facilitate the task of discriminating the peaks from the noise. We applied a smoothing function when preparing the signal before the decomposition but the phasic signal was not completely noise-free.

There are considerations when using the Empatica E4. If the band is too loose, the readings will vary and data will become unreliable. If the band is too tight, it can create discomfort and interfere with the VR experience. Ideally, the signal acquired needs to be as clean as possible to minimise the amount of post-processing. Another consideration that we noticed is that the wristband has a button that needs to be pressed to start and stop recording data. When the button is pressed, the sensors are pressed against the skin of the person wearing it and that is clearly visible in the data with short spikes and oscillation on the first seconds of the recording, as well as at the end. The data needed to be trimmed but, ideally, the best way to operate the wristband is remotely via the API provided by the manufacturer.

Developing VR therapies based on rigorous experiment is more likely to lead to clinically successful outcomes. This study not only confirms the base potential for VR in helping people smoke less but shows that the scenario can form part of the content of a VR therapy.

## Data Availability

Deidentified data are available from the corresponding authors on reasonable request and contract with the university.
